# Diagnostic performance of adult-based ultrasound ACR-TIRADS and C-TIRADS in adolescent thyroid nodules

**DOI:** 10.3389/fendo.2026.1830933

**Published:** 2026-07-01

**Authors:** Jieyu Liu, Chengxue Jiang, Xiaoye Zhang, Taiqing Zheng, Minghui Liu, Guotao Wang

**Affiliations:** 1 Department of Ultrasound, The Second Xiangya Hospital, Central South University, Changsha, China; 2 Department of Pathology, Children’s Hospital, Central South University, Changsha, China

**Keywords:** ACR-TIRADS, C-TIRADS, nodule, pediatric, thyroid

## Abstract

**Objectives:**

This study aims to evaluate the diagnostic performance of the American College of Radiology Thyroid Imaging Reporting and Data System (ACR-TIRADS) and the Chinese Thyroid Imaging Reporting and Data System (C-TIRADS) in differentiating between malignant and benign thyroid nodules in the pediatric population.

**Materials and methods:**

In this retrospective, single-center study, a total of 461 thyroid nodules from 393 pediatric patients (median age, 16.0 years; 91 boys) from September 2018 to November 2024 were analyzed. All diagnostic performance results are reported on a per-nodule basis. Scenario 1 (biopsy recommended for ACR-TR3 nodules ≥35 mm and all ACR-TR5 nodules) and Scenario 2 (biopsy recommended for all C-TR4B, C-TR4C, and C-TR5 nodules, with no biopsy for C-TR4A) were adopted to indicate biopsy. The diagnostic performance, unnecessary biopsy rates, and biopsy triage failure rates of ACR-TIRADS, C-TIRADS, Scenario 1, and Scenario 2 were estimated and compared.

**Results:**

The area under the receiver operating characteristic curve for Scenario 1 and Scenario 2 was significantly higher than that of ACR-TIRADS and C-TIRADS, respectively [0.793 (95% CI: 0.753 -0.829) vs. 0.663 (95% CI: 0.618 -0.706) and 0.825 (95% CI: 0.787 -0.858) vs. 0.689 (95% CI: 0.645 -0.731); both *P* < 0.01] Furthermore, both scenarios demonstrated lower unnecessary biopsy rates (47.5% vs. 60.1% for Scenario 1; 30.6% vs. 56.8% for Scenario 2) and biopsy triage failure rates (5.4% vs. 13.6% for Scenario 1; 8.8% vs. 12.8% for Scenario 2) compared to the corresponding TIRADS systems.

**Conclusions:**

These findings suggest that the application of Scenario 1 and Scenario 2 can improve diagnostic performance and reduce both unnecessary biopsy rates and biopsy triage failure rates in identifying malignant thyroid nodules in the pediatric population.

## Introduction

Although thyroid nodules are less common in children, the incidence of thyroid cancer in the pediatric population has been increasing worldwide in recent years (1, 2). Additionally, pediatric thyroid nodules are more likely to be malignant than adult nodules (19% vs. 12%) (3). Thyroid cancer in children, compared to that in adults, is more likely to present with extrathyroidal extension, metastases, and recurrence (4–6). Therefore, early and accurate differentiation of malignant thyroid nodules is critically important.

As a non-invasive imaging modality, ultrasound (US) is regarded as a useful diagnostic tool for differentiating malignant thyroid nodules from benign ones, which is critical for the clinical management of thyroid nodules (7, 8). To determine whether nodules require fine-needle aspiration (FNA) or follow-up, several US-based risk stratification systems focusing on adult thyroid nodules have been developed by various international societies. These include the American College of Radiology Thyroid Imaging Reporting and Data System (ACR-TIRADS) (9), the Chinese Thyroid Imaging Reporting and Data System (C-TIRADS) (10), the European Thyroid Imaging Reporting and Data System (EU-TIRADS), and the Korean Thyroid Imaging Reporting and Data System (K-TIRADS) (11). These standardized risk-stratification systems have demonstrated adequate diagnostic performance in identifying malignant thyroid nodules in the adult population (12–15). Among them, ACR-TIRADS showed the highest accuracy and the lowest unnecessary biopsy rate, making it more reliable for recommending FNA for thyroid nodules (13, 16, 17). C-TIRADS was regarded as being aligned with China’s national conditions and medical context (10). Both ACR-TIRADS and C-TIRADS have been widely used in the management of adult thyroid nodules in China.

However, the diagnostic performance of ACR-TIRADS and C-TIRADS for identifying malignant thyroid nodules in the pediatric population has not been well evaluated. Pediatric thyroid cancers are known to have different clinical, molecular, and pathological characteristics from those in adults. Therefore, adult-based risk stratification systems may not be entirely appropriate for children (18, 19). Recently, many efforts have been made to apply these US-based risk stratification systems in the pediatric population, but the sample sizes of most studies on TIRADS application for pediatric thyroid nodules have been small (20–23).

In the present study, eligible pediatric patients who underwent thyroid US between September 2018 and November 2024 were retrospectively identified. Two risk-stratification scenarios were applied to identify malignant thyroid nodules: scenario 1 based on ACR-TIRADS and scenario 2 based on C-TIRADS. The aim was to improve diagnostic performance while reducing unnecessary biopsy rates and biopsy triage failure rates.

## Materials and methods

### Patients

**Figure 1:**
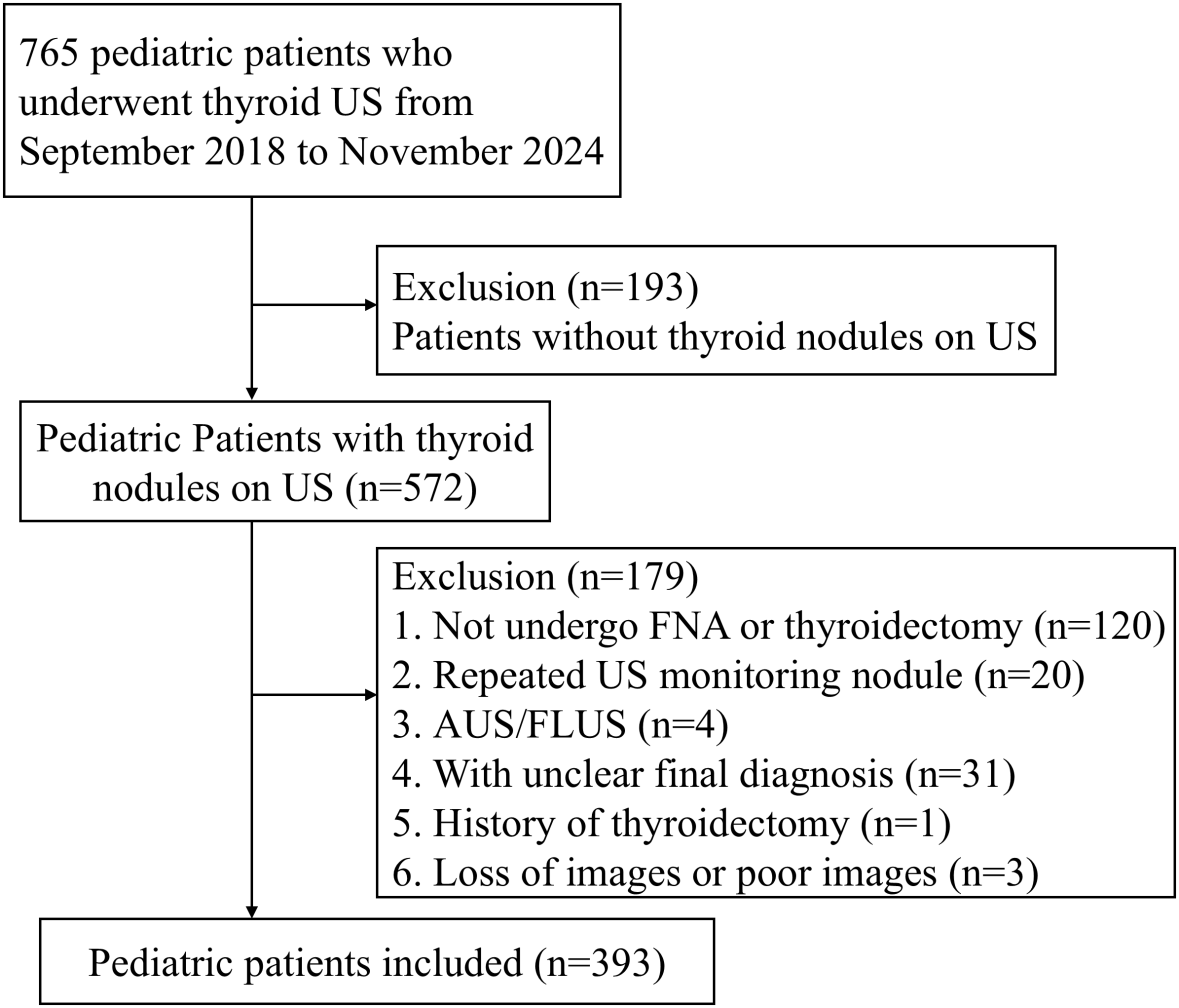
Flowchart of patient selection. *US*, ultrasound; *FNA*, fine-needle aspiration; *AUS*, atypia of undetermined significance; *FLUS*, follicular lesion of undetermined significance.

This retrospective study was approved by the ethics committee of our center, and the need for informed consent was waived. Between September 2018 and November 2024, data from patients who underwent thyroid US were collected. These patients fulfilled the following eligibility criteria: (a) age of 18 years or less, (b) nodules seen with thyroid US, and (c) acceptable diagnostic reference standards. Exclusion criteria for the nodules were as follows: (a) history of thyroidectomy, (b) atypia of undetermined significance or follicular lesion of undetermined significance, (c) an unclear final diagnosis, (d) repeated US monitoring of thyroid nodules, (e) and lost images or poor image quality. The patient selection flowchart is presented in **Figure 1**. The US features of 108 of the 393 patients have been previously reported (24).

The indications for FNA or surgery were established by clinicians based on US findings, nodule size, patient age, symptoms, history of irradiation, cancer predisposition syndromes, and the preference of the patient or legal guardian. Follow-up strategies for nodules without a conclusive histopathologic diagnosis were determined according to the ACR-TIRADS system (9).

**Table 1** shows the comparison of malignancy risk according to each category in the two guidelines. Of the 461 thyroid nodules, 128 (27.8%) were diagnosed as malignant, comprising 122 papillary thyroid carcinomas, 2 follicular carcinomas, 3 medullary carcinomas, and 1 lymphoma. The remaining 333 (72.2%) were diagnosed as benign, including 198 cases of nodular hyperplasia, 96 cases of follicular adenoma, and 39 cases of thyroiditis.

**Table 1:** Comparison of malignancy risk for thyroid nodules according to the ACR- TIRADS and C-TIRADS.

Guideline	Total nodules (n=461)	Benign nodules (n=333)	Malignant nodules (n=128)	Suggested malignancy Risk (%)	Calculated malignancy Risk (%)	*P* Value
ACR-TIRADS						
TR1	22 (4.8)	22 (6.6)	0 (0)	<2	0	
TR2	89 (19.3)	85 (25.5)	4 (3.1)	<2	4.5	
TR3	148 (32.1)	137 (41.2)	11 (8.6)	5	7.4	
TR4	78 (16.9)	56 (16.8)	22 (17.2)	5-20	28.2	
TR5	124 (26.9)	33 (9.9)	91 (71.1)	>20	73.4	<0.001
C-TIRADS						
TR 2	16 (3.4)	16 (4.8)	0 (0)	0	0	
TR 3	158 (34.3)	151 (45.4)	7 (5.5)	<2	4.4	
TR4A	143 (31.0)	122 (36.6)	21 (16.4)	2-10	14.7	
TR4B	88 (19.1)	34 (10.2)	54 (42.2)	10-50	61.4	
TR4C	51 (11.1)	10 (3.0)	41 (32.0)	50-90	80.4	
TR5	5 (1.1)	0 (0)	5 (3.9)	>90	100.0	<0.001

ACR-TIRADS, American College of Radiology Thyroid Imaging Reporting and Data System; C-TIRADS, Chinese Thyroid Imaging Reporting and Data System. Except where indicated, data are numbers of nodules, and numbers in parentheses are percentages.

### Reference standard

The final histopathological diagnosis of benign and malignant nodules was determined by either: (1) cytopathological results from FNA, classified according to The Bethesda System (25); or (2) surgical pathology if thyroidectomy was performed.

### Ultrasound examination and imaging analysis

Thyroid US examinations were performed using an Acuson Sequoia scanner (Siemens Medical Solutions, Mountain View, CA) equipped with a linear array transducer (4–10 MHz), a LOGIQ 9 scanner (GE Healthcare, Milwaukee, WI) equipped with a linear array transducer (10–14 MHz), or a Resona 7 scanner (Mindray, Shenzhen, China) equipped with a linear array transducer (3–11 MHz). The scanning protocol has been detailed in a previous study (24). Key US features of the thyroid nodules, including location, composition, echogenicity, shape, margin, and echogenic foci, were documented by radiologists. All US images were stored in the Picture Archiving and Communication System.

**Figure 2:**
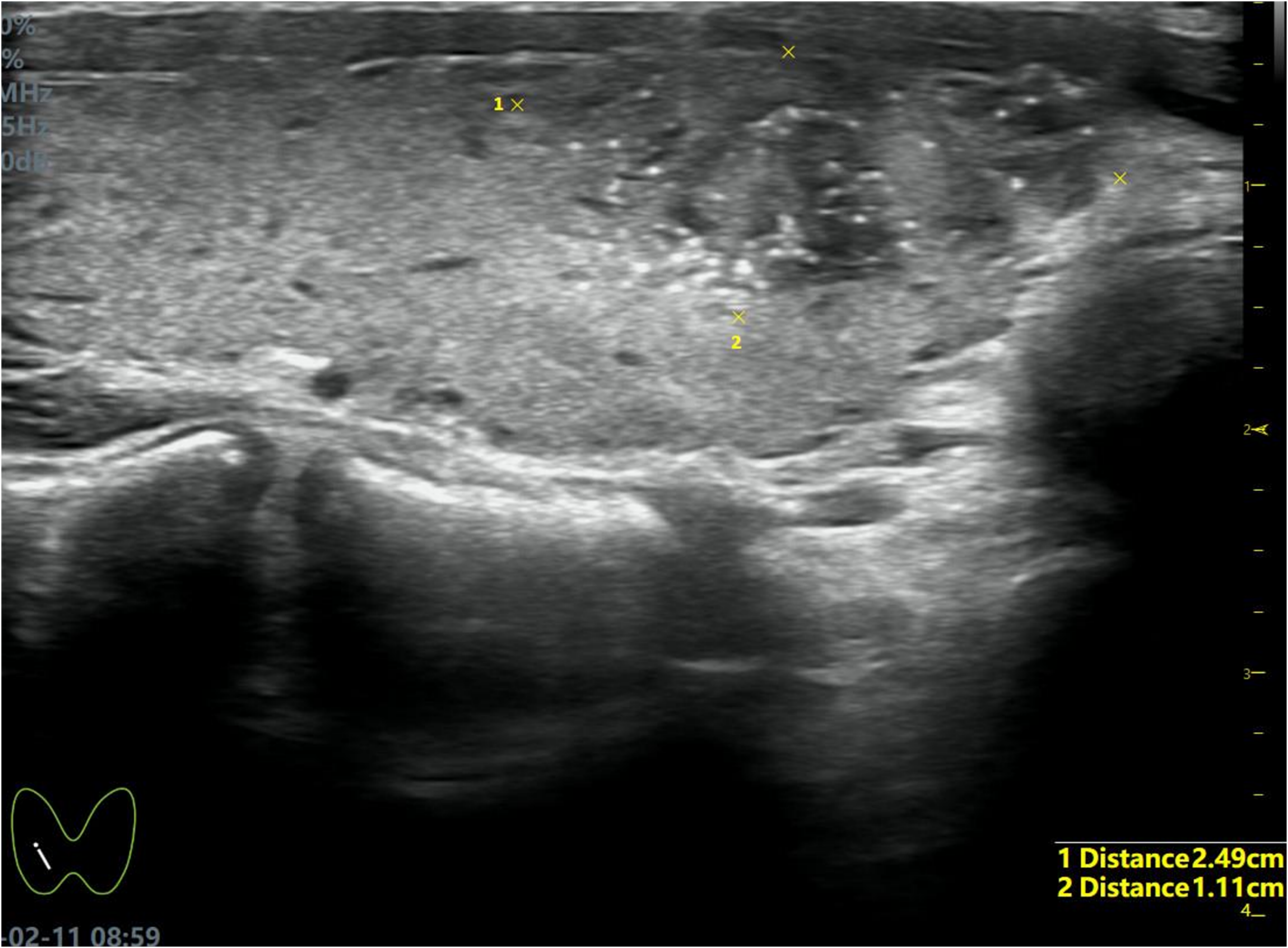
Sagittal gray-scale US image in 14-year-old girl with a papillary cancer shows a 2.5-cm thyroid nodule (calipers) that is solid and hypoechoic, with irregular margins and echogenic foci (C-TR4C).

Two radiologists (L.J.Y. and W.G.T., both with 10 years of experience) who were blinded to other clinical findings randomly reviewed all the US images in consensus. Both radiologists had not been involved in the original examinations. If an inconsistency arose, a third experienced radiologist (L.M.H., with more than 35 years of experience) evaluated the US features to make the final decision. For each nodule, the radiologists were asked to record the primary US image characteristics according to both the 2017 ACR-TIRADS lexicon (9) and the 2020 C-TIRADS lexicon (10), respectively. The definitions of the 2017 ACR-TIRADS lexicon have been introduced in previous study (24). According to the 2020 C-TIRADS, the points of the six US features were as follows: vertical orientation (1 point), solid composition (1 point), markedly hypoechoic (1 point), microcalcifications (1 point), ill-defined/irregular margin or extrathyroidal extension (1 point), and comet artifacts (-1 point). The scores of the above six US characteristics were added to determine the C-TIRADS level. -1 points as C-TR2, 0 points as C-TR3,1 point as C-TR4A, 2 points as C-TR4B, 3-4 points as C-TR4C (**Figure 2**), 5 points as C-TR5, and proved malignant as C-TR6.

### Scenario of size cutoff for FNA indication

Since thyroid volumes in children are smaller than those in adults, the current nodule size cutoffs for FNA recommended by adult guidelines may not be suitable for pediatric patients. Therefore, we established new size-based criteria for FNA indication based on the ACR-TIRADS and C-TIRADS systems. Scenario 1 (ACR-TIRADS) adopted the criteria previously reported (24) : FNA was indicated for TR3 nodules ≥35 mm and for all TR5 nodules. Scenario 2 (C-TIRADS) was defined as follows: FNA was not indicated for TR4A nodules; for nodules in the TR4B, TR4C, and TR5 categories, FNA was indicated for those with a diameter ≥10 mm.

In this study, the unnecessary biopsy rate was defined as the proportion of biopsied nodules that were benign. The biopsy triage failure rate was defined as the proportion of malignant nodules that were not recommended for biopsy.

### Statistical analysis

SPSS software (version 22.0, IBM Corp) and MedCalc software (version 15.2.2, MedCalc Software) were used for statistical analysis. A P value <0.05 (two-sided) was considered statistically significant. Descriptive statistics were expressed as numbers and percentages. Continuous variables were presented as medians and ranges. The Mann–Whitney U test was used for continuous variables and the chi-square test for categorical variables. The diagnostic performance in differentiating benign from malignant thyroid nodules was assessed. Sensitivity, specificity, positive predictive value (PPV), negative predictive value (NPV), area under the receiver operating characteristic curve (AUC), and accuracy were calculated. The AUCs of different risk stratification systems and scenarios were compared using the DeLong test.

## Results

### Patients and thyroid nodules characteristics

The baseline characteristics of the enrolled patients and nodules are summarized in **Table 2**. A total of 461 thyroid nodules from 393 pediatric patients (median age, 16.0 years; 91 boys) met the inclusion criteria and were included in the analysis. The median nodule size was 26.0 mm (interquartile range: 16.0–37.0 mm). Of these, 79 (17.1%) were diagnosed based on cytopathological results from FNA, and 382 (82.9%) were diagnosed by surgical pathology following thyroidectomy.

**Table 2:** The clinical characteristics of 461 thyroid nodules in 393 patients.

Characteristic	Value
No. of patients/nodules	393/461
Age at US (year)*	16.0 (14.0-18.0)
Sex	
Girls	302 (76.8)
Boys	91 (23.2)
Methods of diagnosis	
FNA	79 (17.1)
Surgery	382 (82.9)
Nodule diameter (mm)*	26.0 (16.0-37.0)
Location	
Left lobe	187 (40.6)
Right lobe	260 (56.4)
Isthmus	14 (3.0)

FNA, fine-needle aspiration.

*Data are the median, with the interquartile range in parentheses Except where indicated, data are numbers of patients/nodules, and numbers in parentheses are percentages.

### Malignancy risk according to category in the two guidelines

The malignancy risk for most categories was well aligned with the suggested risk ranges, with the exception of ACR-TR4 (28.2% [22/78]) and C-TR4B (61.4% [54/88]). A higher predicted probability of malignancy was associated with a higher risk category in both guidelines (both *P* < 0.001).

### Diagnostic performance in the prediction of thyroid malignancy

The diagnostic performance of ACR-TIRADS, C-TIRADS, Scenario 1, and Scenario 2 is presented in **Table 3**. For the original ACR-TIRADS, the sensitivity, specificity, PPV, NPV, and accuracy were 77.3% (99/128), 55.3% (184/333), 39.9% (99/248), 86.4% (184/213), and 61.4% (283/461), respectively. When using C-TIRADS to predict malignant thyroid nodules, the sensitivity was 76.6% (98/128), specificity 61.3% (204/333), PPV 43.2% (98/227), NPV 87.2% (204/234), and accuracy 65.5% (302/461).

**Table 3:** Diagnostic performance for diagnosis of malignant thyroid nodules according to the two original guidelines and two scenarios.

Guideline	Sensitivity	Specificity	PPV	NPV	Accuracy	UBR	MMR	AUC	*P* value^a^
ACR-TIRADS	77.3 (69.1 - 84.3)	55.3 (49.7 - 60.7)	39.9 (36.3 - 43.6)	86.4 (82.0 - 89.9)	61.4	60.1	13.6	0.663 (0.618 -0.706)	–
C-TIRADS	76.6 (68.3 - 83.6)	61.3 (55.8 - 66.5)	43.2 (39.2 - 47.3)	87.2 (83.1 - 90.4)	65.5	56.8	12.8	0.689 (0.645 -0.731)	0.077, -
Scenario 1									
ACR-TIRADS	89.8 (83.3 - 4.5)	68.8 (63.5 - 73.7)	52.5 (48.3 - 56.7)	94.6 (91.3 - 96.7)	74.6	47.5	5.4	0.793 (0.753 -0.829)	<0.001, <0.001, -
Scenario 2									
C-TIRADS	78.1 (70.0 - 84.9)	86.8 (82.7 - 90.2)	69.4 (63.0 - 75.2)	91.2 (88.1 - 93.5)	84.4	30.6	8.8	0.825 (0.787 -0.858)	<0.001, <0.001, 0.127

ACR-TIRADS, American College of Radiology Thyroid Imaging Reporting and Data System; C-TIRADS, Chinese Thyroid Imaging Reporting and Data System; UBR, unnecessary biopsy rate; MMR, missed malignancy rate; PPV, positive predictive value, NPV negative predictive value; AUC, area under the curve. Data in parentheses are 95% confidence intervals.

^a^ Only AUCs were compared. The first P-value was compared with ACR-TIRADS guideline, the second P-value was compared with C-TIRADS guideline, the third P-value was compared with scenario 1.

Using Scenario 1 (ACR-TIRADS: FNA for TR3 nodules ≥35 mm and all TR5 nodules) to predict thyroid malignancy, the sensitivity, specificity, PPV, NPV, and accuracy were 89.8% (115/128), 68.8% (229/333), 52.5% (115/219), 94.6% (229/242), and 74.6% (344/461), respectively. When Scenario 2 (C-TIRADS: no FNA for TR4A nodules, and FNA for all TR4B, TR4C, and TR5 nodules) was used, the sensitivity, specificity, PPV, NPV, and accuracy were 78.1% (100/128), 86.8% (289/333), 69.4% (100/144), 91.2% (289/317), and 84.4% (389/461), respectively.

The AUCs of Scenario 1 and Scenario 2 were significantly higher than those of the original ACR-TIRADS and C-TIRADS (all P < 0.001). No significant difference was observed between the diagnostic performance of Scenario 1 and Scenario 2 (*P* = 0.127) (**Table 3**). In addition, Scenario 1 yielded the lowest biopsy triage failure rate (Scenario 1: 5.4% [13/242]; Scenario 2: 8.8% [28/317]; ACR-TIRADS: 13.6% [29/213]; C-TIRADS: 12.8% [30/234]), while Scenario 2 achieved the lowest unnecessary biopsy rate (Scenario 2: 30.6% [44/144]; Scenario 1: 47.5% [104/219]; ACR-TIRADS: 60.1% [149/248]; C-TIRADS: 56.8% [129/227]).

Subgroup analysis showed that although the malignancy rate was significantly higher in the surgery-confirmed group than in the FNA-confirmed group (31.2% vs. 11.4%, P < 0.001), the AUCs of both modified scenarios (Scenario 1 and Scenario 2) did not differ significantly between the two subgroups (*P* = 0.780 and *P* = 0.377, respectively).

In the surgical histopathology subgroup (n=382), the diagnostic performance of Scenario 1 (AUC: 0.803, 95%CI: 0.759 - 0.842) and Scenario 2 (AUC: 0.845, 95%CI: 0.804 - 0.880) remained superior to the original ACR-TIRADS (0.668, 95%CI: 0.619 - 0.715) and C-TIRADS (0.704, 95%CI: 0.656 - 0.750), respectively (**Supplementary Table 1**), consistent with the main analysis.

## Discussion

In this study, ACR-TIRADS and C-TIRADS were applied to the pediatric population for discriminating malignant thyroid nodules. Scenario 1, based on ACR-TIRADS (FNA for TR3 nodules ≥35 mm and all TR5 nodules), was used as previously reported (24). In addition, Scenario 2, based on C-TIRADS (FNA for all TR4B, TR4C, and TR5 nodules, with no FNA for TR4A), was adopted to indicate FNA for predicting thyroid malignancy in pediatric patients.

The AUCs of Scenario 1 and Scenario 2 were significantly higher than those of ACR-TIRADS and C-TIRADS (Scenario 1 vs. ACR-TIRADS: 0.793 (95% CI: 0.753 -0.829) vs. 0.663 (95% CI: 0.618 -0.706), *P* < 0.001; Scenario 2 vs. C-TIRADS: 0.825 (95% CI: 0.787 -0.858) vs. 0.689 (95% CI: 0.645 -0.731), *P* < 0.001). Correspondingly, both Scenario 1 and Scenario 2 achieved lower unnecessary biopsy rates (47.5% vs. 60.1%; 30.6% vs. 56.8%) and biopsy triage failure rates (5.4% vs. 13.6%; 8.8% vs. 12.8%) compared to the original guidelines. No significant difference was observed between the diagnostic performance of Scenario 1 and Scenario 2 for predicting thyroid malignancy (0.793 vs. 0.825, *P* = 0.127), indicating that both scenarios demonstrated satisfactory diagnostic performance in differentiating malignant from benign thyroid nodules.

According to previous reports, the malignancy rates of thyroid nodules in the pediatric population vary widely, ranging from 13.0% to 46.6% (22, 26, 27). In the present study, the overall malignancy rate was 27.8% (128/461). Notably, the malignancy rates in the highest categories of ACR-TIRADS and C-TIRADS were 73.4% and 100%, respectively. The 100% malignancy rate observed for C-TR5 may be attributed to the relatively small sample size in this category. Importantly, both Scenario 1 and Scenario 2 demonstrated higher sensitivity (89.8% vs. 77.3% and 78.1% vs. 76.6%) and higher specificity (68.8% vs. 55.3% and 86.8% vs. 61.3%) compared to the original ACR-TIRADS and C-TIRADS, respectively. These findings suggest that modifying the size thresholds for biopsy in pediatric patients can improve the diagnostic performance for differentiating malignant thyroid nodules.

For nodules classified as C-TR4B, C-TR4C, and C-TR5, FNA may be recommended in children regardless of nodule size. Because the thyroid volume in the pediatric population is smaller than that in adults, nodules smaller than 10 mm in these categories should not be ignored. Fu et al. also suggested that biopsying all nodules in the C-TR4B, C-TR4C, and C-TR5 categories may be an effective strategy (28). Their modified C-TIRADS achieved a lower unnecessary biopsy rate (38.3% vs. 52.2%) and biopsy triage failure rate (7.7% vs. 44.9%) compared to the original C-TIRADS, which is comparable to our findings in pediatric patients (30.6% vs. 56.8% and 8.8% vs. 12.8%, respectively).

According to the C-TIRADS, the malignancy rate for C-TR4A is relatively low (2–10%) (10). In the present study, 31% (143/461) of nodules were classified as C-TR4A, with a malignancy rate of 14.7%, indicating that approximately 85% of C-TR4A nodules were benign. Since thyroid nodules with solid composition are categorized as C-TR4A, applying the standard FNA criteria would lead to a large number of unnecessary biopsies in pediatric patients. For young children, parents may be reluctant to consent to FNA, as sedation or general anesthesia is often required. Therefore, only selected patients with a higher probability of malignancy underwent biopsy in our study. To avoid unnecessary procedures, FNA is not recommended for C-TR4A thyroid nodules in the pediatric population based on our findings. The modified FNA criteria (≥35 mm for ACR-TR3 and all ACR-TR5 nodules) improved diagnostic performance while reducing unnecessary biopsy rates and biopsy triage failure rates in children. Our previous study (24) also confirmed the satisfactory diagnostic performance of these modified criteria for FNA.

C-TIRADS may offer a more scientific, simpler, and clearer approach than other TIRADS versions for the Chinese population. It has demonstrated superior discriminatory performance compared to ACR-TIRADS in distinguishing benign from malignant thyroid nodules in Chinese adults (29, 30). However, its diagnostic performance in the pediatric population has not been well evaluated. Our results demonstrate that the modified C-TIRADS achieves comparably satisfactory performance to the modified ACR-TIRADS in malignancy risk stratification of thyroid nodules in children. Notably, Scenario 1 (modified ACR-TIRADS) yielded a lower biopsy triage failure rate, whereas Scenario 2 (modified C-TIRADS) achieved a lower unnecessary biopsy rate. Therefore, these modified guidelines based on C-TIRADS and ACR-TIRADS may be more suitable for managing thyroid nodules in pediatric patients. Scenario 1 prioritized sensitivity (89.8%) at the cost of a higher unnecessary biopsy rate (47.5%), whereas Scenario 2 achieved a lower unnecessary biopsy rate (30.6%) with slightly lower sensitivity (78.1%). The choice between scenarios depends on clinical priorities.

The present study has several limitations. First, our study only included thyroid nodules with a confirmed pathological diagnosis. Although this inclusion criterion is required for diagnostic accuracy studies, it inevitably introduces spectrum bias. Compared with consecutive ultrasound-based screening cohorts, the malignancy rate in our cohort is overestimated, which may lead to overestimation of sensitivity and positive predictive value. Therefore, our diagnostic performance metrics, particularly PPV, should not be directly extrapolated to general pediatric screening populations. Second, this was a single-center retrospective study with a relatively small sample size; therefore, our findings require further validation in other centers. Third, this study was conducted at a single center in China, and the C-TIRADS system is region-specific to the Chinese population. Therefore, the generalizability of our findings to non-Chinese populations or healthcare systems with different clinical pathways and biopsy practices may be limited. Future multi-center, international studies are needed to validate the proposed scenarios in diverse pediatric populations. Fourth, other US risk stratification systems, such as EU-TIRADS and K-TIRADS, were not evaluated in our study.

In conclusion, applying modified biopsy criteria—Scenario 1 (FNA for ACR-TR3 nodules ≥35 mm and all ACR-TR5 nodules) and Scenario 2 (FNA for all C-TR4B, C-TR4C, and C-TR5 nodules, with no FNA for C-TR4A)—improved diagnostic performance for identifying malignant thyroid nodules in pediatric patients. Both scenarios reduced unnecessary biopsy rates and biopsy triage failure rates compared to the original guidelines.

## Data Availability

The raw data supporting the conclusions of this article will be made available by the authors, without undue reservation.
