# Supplementary material for: Diagnostic performance of adult-based ultrasound ACR-TIRADS and C-TIRADS in adolescent thyroid nodules
Source: Front Endocrinol (Lausanne). 2026 Jul 1;17:1830933. doi: 10.3389/fendo.2026.1830933 (PMC13368548; doi:10.3389/fendo.2026.1830933)
Supplement: Supplementary file 1 [file Table1.docx]

Supplementary Table 1: Diagnostic performance for malignant thyroid nodules according to the two original guidelines and two scenarios in the 382 nodules with surgical histopathology as the reference standard

| Guideline | Sensitivity | Specificity | PPV | NPV | AUC |
| --- | --- | --- | --- | --- | --- |
| ACR-TIRADS | 78.2  (69.6 - 85.2) | 55.5  (49.3 - 61.6) | 44.3  (40.3 - 48.4) | 84.9  (79.7 - 88.9) | 0.668  (0.619 - 0.715) |
| C-TIRADS | 78.2  (69.6 - 85.2) | 62.7  (56.6 - 68.6) | 48.7  (44.1 - 53.3) | 86.4  (81.7 - 90.0) | 0.704  (0.656 - 0.750) |
| Scenario 1 |  |  |  |  |  |
| ACR-TIRADS | 89.1  (82.0 - 94.1) | 71.5  (65.6 - 76.9) | 58.6  (53.6 - 63.4) | 93.5  (89.6 - 96.0) | 0.803  (0.759 - 0.842) |
| Scenario 2 |  |  |  |  |  |
| C-TIRADS | 77.3  (68.7 - 84.5) | 91.6  (87.6 - 94.7) | 80.7  (73.5 - 86.3) | 89.9  (86.5 - 92.6) | 0.845  (0.804 - 0.880) |

ACR-TIRADS American College of Radiology Thyroid Imaging Reporting and Data System, C-TIRADS Chinese Thyroid Imaging Reporting and Data System, PPV positive predictive value, NPV negative predictive value, AUC area under the curve

Data in parentheses are 95% confidence intervals
